# Supplementary material for: Are carbon emissions trading and green financial instruments synergistic? -Comprehensive quantitative research based on content analysis
Source: PLoS One. 2024 Mar 7;19(3):e0298601. doi: 10.1371/journal.pone.0298601 (PMC10919601; doi:10.1371/journal.pone.0298601)
Supplement: S1 Data — (ZIP) [file pone.0298601.s001.zip › Data/地级市Z_F工作报告.zip!/─┌├╔╣┼/║⌠┬╫▒┤╢√╩╨/└┤╫╘╣1⁄2╓┌║┼í╛┬φ┐╦╩2╛▌═°í┐_2011.docx]

各位代表：

现在，我代表呼伦贝尔市人民政府向大会报告工作，请予审议，并请市政协委员和列席会议的同志提出意见。

一、“十一五”时期经济社会发展回顾

“十一五”时期，是我市发展进程中极不平凡的五年。在自治区党委、政府和市委的正确领导下，我们坚持美丽与发展双赢战略，团结带领全市各族人民，积极应对金融危机等诸多困难和挑战，提前一年完成了“十一五”规划各项目标任务，开创了全市改革开放和现代化建设的新局面。

综合经济实力显著增强。五年来，我们以“五区建设”为重点，抢抓机遇，乘势而上，实现了经济又好又快发展。地区生产总值达到932亿元，年均增长16.1％。财政总收入完成124.68亿元，其中，地方财政总收入完成93.22亿元，年均增长24.7％；地方财政总支出达到207.9亿元，年均增长29.7%。全社会固定资产投资累计完成2039.8亿元，是“十五”时期的3.7倍。农牧业基础地位进一步巩固，发展质量和效益全面提升，全市粮食产量突破百亿斤，跨入全国20个百亿斤产粮大市行列；牧业年度牲畜存栏达到1610万头只。第三产业快速发展，加快推进呼伦贝尔民族文化园、白音哈达草原景区、蒙古之源•蒙兀室韦和拓跋鲜卑民族文化园等景区建设，旅游航线由4条增加到28条，年进出港旅客增长4.7倍，旅游总收入达到143亿元，增长3.1倍；口岸基础设施建设进一步完善，外贸进出口总额累计完成122亿美元，口岸进出境货运量达到2632万吨，正式开通了呼伦贝尔国际电子商务应用平台；保险、物流等现代服务业实现了长足发展，银行存款总量年均增长26.8%，达到762.3亿元，贷款额度年均增长34.7%，达到430.5亿元。我市先后获得“CCTV2006--中国最佳民族风情魅力城市”、“国际旅游名城”、“中国人居环境范例奖”等殊荣。

经济结构进一步优化。注重经济结构调整，促进三次产业协调发展。全力推进新型工业化进程，引进了国网公司、华能、大唐、华电、神华、中国黄金、江苏雨润、瑞士雀巢、云南驰宏、东方希望等40多家国内外知名大企业，规模以上工业企业由167户增加到427户；初步构建了煤炭、电力、化工、农畜产品加工、石油和有色金属等十大产业集群。战略性新兴产业和非资源型产业开始成长，风力发电、装备制造、生物制药、电子信息等项目扎实推进。非公有制经济占全市GDP的比重达到50.1％。三次产业由2005年的27.5：30.5：42.0调整为19.6：42.1：38.3，其中，全部工业增加值年均增长24.4%，工业占经济总量的比重由23.9%提高到36.2%。经济结构进一步优化。

改革开放继续推进。强化改革举措，着力破解体制机制的制约。呼伦贝尔电网整体划归国家电网，大雁、宝日希勒、扎赉诺尔三大煤业公司成功实现重组，森工、铁路、农垦、地方林业6局等企业剥离办社会和主辅分离改革取得重大突破。农村牧区经营体制改革有序开展，全市流转土地710万亩，流转草场900万亩，各类农牧民专业合作经济组织达到1844个。集体林权制度改革深入推进。包商银行、内蒙古银行和浦发银行等金融机构先后入驻我市，农村信用社产权制度改革基本完成。医药卫生体制改革启动实施，文化体制改革逐步推开。实施绩效工资改革，以岗位管理和聘用制为重点的事业单位人事制度改革进一步深化。国际交流合作领域进一步拓展，中俄东段划界和中蒙两个口岸联检楼建设顺利完成，满洲里被国家列为重点开发开放试验区。主动融入东北老工业基地，积极承接发达地区产业转移，五年累计引进市外（国内）资金1644亿元，是“十五”时期的6.2倍；实际利用外资4.3亿美元，是“十五”时期的4.2倍，要素流入势头和经济外向度不断增强。

发展条件明显改善。把生态作为最大的基础设施来抓，五年累计投入资金17.37亿元，实施了天保工程、重点公益林保护、退耕还林还草等工程。国家级生态市创建扎实推进，国家森林城市创建开始启动，累计退耕542.6万亩、退牧2520万亩，活立木蓄积量增加了9154万立方米，森林覆盖率达到51.25%；特别是近两年，实施了大规模沙区综合治理工程，累计投资3.1亿元，治理沙地210万亩。单位地区生产总值能源消耗和主要污染物减排总量均完成了自治区下达的指标任务。加快推进城镇基础设施建设，五年累计投入115亿元，实施了市政道路、供水供热、污水垃圾处理等项目1026个；投资315亿元，房屋建设总面积达到1978万平方米，其中住宅面积1510万平方米，全市城镇化率达到了67.5%；启动实施了市中心城新区、满洲里新区、阿荣旗新区建设，市中心城新区市直机关业务用房、市体育馆、哈萨尔斜拉桥、满洲里新国门、呼伦贝尔大剧院等一批标志性建筑投入使用。公路建设五年累计完成投资193.7亿元，新建公路9250公里，开工建设了绥满国道主干线阿荣旗-博克图-牙克石段高速公路和海拉尔-阿木古郎-杜拉尔桥一级公路；完成了海拉尔机场路和北出口景观大道建设；实现了所有乡镇(苏木)通油路、行政村(嘎查)通公路的目标。铁路五年累计完成投资53.4亿元，建设总里程431公里，两伊铁路竣工通车，滨洲铁路复线全线贯通，开工建设了阿荣旗-扎兰屯地方铁路。呼伦贝尔海拉尔机场航站楼改扩建和机场跑道扩建、满洲里新机场建成投入使用。呼伦贝尔－辽宁50万伏直流输电工程全面竣工，电力外送能力进一步增强。资源勘查开发成效显著，矿产资源总体规划基本完成。一批重点水利工程相继发挥作用，尼尔基水库全面建成，红花尔基水库建成蓄水发电，呼伦湖水资源配置及水环境治理工程生态效益初显；农村牧区安全饮水工程扎实推进，解决了53.9万人的安全饮水问题；市中心城区给水改扩建工程全面完成。

人民生活水平持续提高。以“六个一”幸福指数工程为统领，全面加大改善民生力度，五年累计投入资金375.7亿元，是“十五”时期的3.5倍，重点实施了46个为民办实事项目和41项公共公益工程，解决了就医就学、住房出行、用水用电、生态环境等一大批事关群众切身利益的问题，人民群众的幸福感明显增强。全市城镇居民人均可支配收入达到14857元，五年增加6629元；农牧民人均纯收入达到6295元，五年增加3093元。投资100亿元，建设保障性住房669万平方米，惠及12万户家庭，全市人均住房面积达到29平方米。城镇累计新增就业13.7万人，城镇登记失业率控制在4.12%以内。健全了覆盖城乡居民的社会保障体系，各项社会保险待遇逐年提高，全市基本养老、失业、医疗、工伤、生育保险参保人数分别达到42.8万人、30.99万人、130.09万人、20.48万人和22.08万人。开创性地实施了新农保试点工作，参保人数达到18.23万人。新农合参保人数达63.2万人，参合率97.3%。城市低保月均补助达到231元，农村牧区低保年均补助水平提高了11.3%。残疾人事业长足发展，对弱势群众的扶助力度全面加大。光荣院、儿童福利院、社会福利院等爱心家园项目相继投入使用。扶贫开发成效明显，累计解决了62.1万贫困人口的温饱问题。自治区帮扶鄂伦春旗和莫旗工作扎实推进，累计投入11.9亿元，实施项目460个，少数民族群众生产生活条件得到明显改善。

社会事业全面进步。深入实施科教兴市战略，30项科技成果获得自治区科学技术进步奖，科技创业服务中心项目稳步推进。财政用于教育支出98.7亿元，是“十五”时期的3.3倍，各级各类教育办学条件明显改善，办学质量显著提高，中小学校舍安全工程全面推进，市职业技术学院、市特教中心和内蒙古大学满洲里学院建成投入使用。倾力打造民族文化品牌，《五彩呼伦贝尔儿童合唱》、《天骄•成吉思汗》等五张文化名片进一步提升了地区影响力，各类博物馆达到37个。农村牧区三级医疗卫生服务体系逐步完善，突发公共卫生事件应急机制进一步健全，有效防控了甲型H1N1流感等重大传染病疫情，市蒙医医院和市结核病院住院楼建成投入使用，市精神卫生中心开工建设。人口和计划生育工作又有新进展。人才队伍建设和智力引进工作取得实效。全市广播电视覆盖率分别达到93.8%和93.9%。全市行政村（嘎查）通信网络覆盖率超过90%。双拥工作成效显著，国防后备力量建设取得新成绩，荣获“全国双拥模范城”称号。统计、粮食、供销、质监、物价、信息化、人防、体育、气象、地震、老龄、残联、邮政、工商联、档案史志、红十字会、妇女儿童和对外联络等工作都有新进展。

民主法制建设不断加强。自觉接受人大依法监督、政协民主监督和各民主党派、无党派人士以及社会各界的舆论监督，累计办理人大代表议案7件，意见、批评和建议738件，政协委员提案983件。重视发挥共青团、妇联等群众团体作用，深入开展政务、厂务和村务公开，扩大了群众的知情权和参与权。扎实开展“三抓一建”活动，有效解决了机关干部思想、作风和纪律中存在的突出问题。强化法制意识，加强审计监督和普法教育，行政执法和公正司法水平逐步提高。认真落实民族宗教政策，社会主义新型民族关系继续巩固。全面开展“平安呼伦贝尔”创建活动，社会治安防控体系更加完善。深入开展基层矛盾排查化解活动，大信访、大调解格局基本形成。进一步加强安全生产监管，安全生产形势总体平稳。全市民族团结、社会稳定、边疆安宁的大好局面得以巩固。建立健全政风行风评议制度，发展环境不断优化。精神文明建设成果丰硕，以会促创，成功举办了五届全市两个文明建设经验交流会，承办了全区第三届新农村新牧区精神文明建设经验交流会，开展了两届道德模范评选表彰活动，“博大、至诚、和美、共赢”的城市精神深入人心。满洲里市荣获“全国文明城市”称号。

各位代表，“十一五”时期是我市综合经济实力提升最快、城乡面貌变化最大、社会建设成就最好、人民群众得到实惠最多的时期之一。我们在全面建设小康社会的道路上迈出了重要步伐，为“十二五”经济社会又好又快发展奠定了更加坚实的基础。这些成绩的取得，是自治区党委、政府和市委正确领导的结果，是市人大、政协有效监督和大力支持的结果，是全市各族人民团结奋斗的结果，是社会各界关心和帮助的结果。在此，我代表市人民政府，向全市各族人民，向为呼伦贝尔建设和发展作出突出贡献的同志们，向关心和支持呼伦贝尔的朋友们，向驻市军警部队指战员，表示衷心的感谢和崇高的敬意!

在肯定成绩的同时，我们也要清醒地认识到发展中存在的矛盾与不足：我市欠发达这个基本市情还没有根本改变，经济总量小，财政收支困难，发展不足仍然是主要矛盾；经济结构不合理，产业结构单一，可持续发展能力不强；经济增长的政策、环境约束强化，固定资产投资和项目建设难度加大；生态环境脆弱，生态保护与建设仍然任重道远；基础设施建设滞后，历史欠帐多，交通网络、电力外送和农田水利设施等瓶颈急需破解；改善民生任务繁重，提高基本公共服务水平压力加大；地区发展不够协调，有的地区缺乏新的经济增长点，产业培育和经济转型任务艰巨；长期积累下来的制约科学发展的体制机制障碍依然存在，容易引发社会矛盾的历史形成的国企改革等遗留问题还没有完全解决，社会稳定和社会管理仍需加强；部分干部的思想观念、工作作风和执行能力有待进一步转变和加强。这些，需要通过我们的艰苦工作和不懈努力，切实加以解决。

二、“十二五”经济社会发展的目标任务

“十二五”时期，是我市加快发展的关键时期，也是调整经济结构、转变发展方式的攻坚时期。根据市委二届十六次全委会议精神，“十二五”时期全市经济社会发展的总体要求是：以科学发展观为指导，紧紧抓住发展不足这个主要矛盾，以美丽发展、科学崛起、共享繁荣为主旋律，以推进新型工业化、城镇化、农牧业现代化为主攻方向，以加强“五区”建设为重要支撑，以深化改革、扩大开放、营造和谐稳定社会环境为持续动力，全力做大经济总量，大力优化经济结构，努力转变发展方式，切实加强基础设施建设，继续保持经济社会良好的发展态势，全面完成“十二五”发展的目标任务，建设幸福的呼伦贝尔。

“十二五”时期全市经济社会的主要发展目标是：地区生产总值年均增长18%，地方财政总收入年均增长18%，固定资产投资年均增长25%，城乡居民收入年均增长17%和15%，到2015年，以上指标均实现翻番增长。节能减排等约束性指标达到自治区要求，生态市建设内容基本完成。人口自然增长率控制在5‰以内，城镇登记失业率控制在4.2%以下。

中央综合判断国际国内形势，认为我国发展仍处于大有作为的重要战略机遇期。具体分析我市“十二五”面临的发展形势，应该说有机遇、有挑战，机遇大于挑战。只要我们认真贯彻落实胡锦涛总书记在十一届全国人大四次会议内蒙古代表团审议时的讲话精神，抢抓发展机遇，突出发展重点，明确发展任务，脚踏实地，奋力拼搏，就一定能够实现我们的目标任务，将呼伦贝尔打造成为国家大型优势特色产业基地、北疆生态安全屏障、沿边开发开放试验区、国际原生态多民俗旅游胜地、边疆民族和谐幸福家园。

在今后的发展中我们必须做到三个坚持：一是坚持美丽与发展并重。生态良好是呼伦贝尔的最大优势、最大资源，是我们生存和发展的基础。必须把生态建设与保护放在优先地位，在保护中发展，在发展中保护，用最小的开发节点，取得最大的经济效益，不断提高可持续发展能力。二是坚持做大总量与结构调整并重。我市资源丰富，发展潜力巨大，具备三次产业协调发展的优势和做大做强的条件。“十二五”时期必须把做大经济总量作为首要任务，以结构调整为主线，全力加快工业化、城镇化和农牧林业现代化进程，着力提升资源型产业层次，着力在农畜产品加工转化上寻求突破，着力提升服务业发展水平，构建合理的经济结构，实现三次产业协调可持续发展。三是坚持发展经济与改善民生并重。我们所做的一切，目的都是为全市各族人民谋福祉。在努力推进经济发展的进程中，要同步提高人民收入水平。在具体工作安排上，要拿出更多财力、物力和精力，用于民生事业和社会事业发展，尤其要高度关注弱势群体，缩小贫富差距，推进共享繁荣。

为此，我们要认真做好以下工作：

(一)务必保持良好发展势头。保持经济良好发展势头，“十二五”期间必须在投资、项目和调整结构方面狠下功夫。一要狠抓投资。投资是拉动全市经济增长的主要动力。要深入研究落实好国家近期密集出台和将要出台的一系列政策措施，努力争取上级投资。加大招商引资力度，积极承接产业转移，吸引更多资金流入。加强政策引导和扶持，鼓励支持本市民间资本投入到经济社会建设的各个领域。二要狠抓项目建设。既要抓立市的大项目，又要抓配套的中小项目；既要抓对经济支撑能力强的项目，又要抓吸纳就业的劳动密集型项目；既要抓已落地的项目建设，又要抓新项目的储备和谋划，努力提高项目建设的质量和效益。三要狠抓结构调整。坚持在增量中调整结构、在转型中优化升级，依托大企业，实施大项目，建设大基地，促进资源型产业、非资源型产业和战略性新兴产业协调发展。集中打造一批产值超百亿的大型企业集团和龙头工业园区，构筑结构优化的新型工业体系。全力推进农牧林业现代化建设，以增产、增效、增收、增绿为目标，构建以粮、油、豆、薯、乳、肉、草等农畜产品为主的种养加产供销一体化产业链，大力引进培育龙头企业，提升产业层次，提高农畜产品加工转化率。以打造国际原生态多民俗旅游胜地和联通俄蒙、对接东北的区域物流中心为目标，积极发展现代服务业。

(二)大力改善发展条件。一要加强生态保护与建设。以生态市建设为目标，实施“集中收缩”战略，促进人口向城镇集中、工业向园区集聚。坚持“面上保护”原则，以保护优先和自然恢复为主，组织实施天保二期等各类重点生态工程。落实草原生态保护补助奖励政策，继续开展大规模的治沙行动。创新资源开发利用模式，大力发展低碳、循环和绿色经济，强化节能减排工作。充分发挥碳汇优势，把我市打造成国家重要的森林草原碳汇基地。二要加强基础设施建设。统筹铁路、公路和航空综合交通运输网建设，加快推进快速铁路客运通道前期、铁路煤运通道、出口高速公路、旗市通一级公路、支线通勤机场群建设，提高市内公路通达深度和公路等级，构建连通俄蒙、畅通周边、覆盖城乡的综合交通运输网。进一步加大工业园区建设力度，提高承载工业经济发展能力。加快推进农网改造、电网主网架建设和电源外送通道、化工产品输送管道建设，解决外送通道制约瓶颈。加强农田水利和大型水利枢纽建设，加快中小河流治理和小型水库除险加固及水资源配置工程建设。基本解决农牧林区安全饮水问题。加强信息基础设施建设，大力发展数字化城市。三要加快城镇化进程。着力做大做强呼伦贝尔中心城区，加快发展副中心城市、旗城关镇和重点镇。做好城市总体规划修编和控制性详细规划编制工作，统筹推进工业园区与城镇建设，实现工业化与城镇化互动发展。建立以工促农、以城带乡长效机制，推动城镇基础设施向农村牧区延伸，公共服务向农村牧区覆盖，做大中心城镇，增强城镇的辐射带动功能。

(三)着力深化改革扩大开放。一要深化体制机制改革。支持森工、农垦等国有大企业深化改革，打造具有竞争优势的大型企业集团。深化农村牧区综合改革，进一步完善土地、草牧场使用权流转制度。加快财政体制改革，完善预算编制，加强执行管理。加快社会事业改革，稳步推进事业单位分类改革，引入竞争机制，更好地满足群众的多样化需求。二要加强区域合作。加强与东部盟市合作，积极融入东北老工业基地，推动区域经济一体化发展。主动承接发达地区产业转移，吸引先进地区生产要素向我市流动。三要扩大对外开放。支持满洲里国家重点开发开放试验区建设，积极争取将呼伦贝尔整体纳入国家开发开放试验区。充分利用国内外“两种资源”、“两个市场”，积极打造口岸合作平台，建立合作共赢的区域合作新机制，努力提高开放水平。

(四)切实保障和改善民生。大力提高城乡居民收入水平。实施更加积极的就业政策，推动劳动就业，支持创业带动就业，扩大就业容量。落实国家增资政策和工资集体协商制度，健全企业职工工资正常增长机制和支付保障机制，逐步提高劳动报酬，实现城乡居民收入和经济发展同步增长。加快推进覆盖城乡居民的社会保障体系建设，在扩大范围、提高保障水平和统筹层次等方面迈出更大的步伐。继续加大扶贫开发力度，确保贫困农牧民稳定增收。加快保障性安居工程建设，确保完成廉租住房、公租住房、经济适用住房、棚户区改造、农村牧区危房改造和游牧民族定居工程等建设任务。加快社会事业发展。不断加大科技投入力度，形成多元化、可持续的投入体系，加强科技攻关、成果转化和人才队伍建设，提高自主创新能力。加大教育投入，财政性教育经费支出占GDP的比重达到4%以上，巩固“普九”成果，推动各级各类教育协调发展，促进教育公平。以农村牧区为重点加强公益性文化基础设施建设，基本建成公共文化服务体系；深化文化体制改革，加快文化产业发展，建设民族文化大市。加快医药卫生事业改革，实施国家基本药物制度，逐步推进公立医院改革，健全覆盖城乡居民的基本医疗卫生服务体系，强化公共卫生服务，支持蒙中医药事业发展。加强食品药品安全监管，确保群众饮食、用药安全。加强人口和计划生育工作，稳定适度低生育水平，提高人口素质，改善人口结构。贯彻落实《全民健身计划》，提高体育竞技水平和全民身体素质。

（五）全力维护社会稳定。统筹协调各方利益关系，坚决制止和纠正损害群众利益的行为，维护社会公平正义。健全社会矛盾排查化解长效机制，有效预防和妥善处置群体性事件和突发性公共事件。完善社会治安防控体系，加强社会治安综合治理，依法严厉打击敌对势力和各种犯罪活动，营造安全的社会环境。认真落实安全生产责任制，遏制重特大安全事故，确保人民群众生命财产安全。全面贯彻党的民族宗教政策，促进各民族团结。支持国防、边防和军队建设，巩固发展良好的军政军民关系，筑牢祖国北疆安全稳定屏障。

三、2011年主要工作

2011年是“十二五”的开局之年，我们将迎来中国共产党成立九十周年，做好今年的工作意义重大。今年全市经济社会发展的主要预期目标是：地区生产总值增长18%，地方财政总收入增长18%，固定资产投资增长25%，城镇居民人均可支配收入增长17%，农牧民人均纯收入增长15%。城镇登记失业率控制在4.15%以内。人口自然增长率控制在5‰以下。全面完成自治区下达的节能减排等约束性指标任务。

为实现上述目标，今年重点抓好8项工作：

（一）以项目和园区建设为载体，做大工业经济总量。实施市级工业重点项目88个，工业固定资产投资完成440亿元，增长21%。规模以上工业增加值增长24%。一要做大做强优势特色产业。以构筑大企业、大产业、大基地为目标，大力发展煤炭、电力、化工、农畜产品加工和有色金属等优势特色产业，促进各产业集群化发展。大力推动大唐谢尔塔拉年产700万吨露天矿、国网能源鄂温克2×60万千瓦电力、驰宏矿业20万吨铅锌冶炼、云天化年产50万吨合成氨80万吨尿素、福润莫旗年加工150万头生猪等项目达产达效；确保华电2×600万吨褐煤多联产、呼兴电网升级改造、华能牙克石2×20万千瓦热电、雨润阿荣旗年加工10万头肉牛、雀巢额尔古纳综合扩能改造等项目开工建设；积极争取呼伦贝尔—山东 800千伏特高压外送通道工程和巴彦哈达、五一牧场等煤田开发列入国家“十二五”专项规划并尽早开工；抓好有色金属探矿权整合工作，制定倾斜政策，鼓励有色金属探采矿权向大型冶炼加工企业流转，实现采选冶加一体化。二要加快培育战略性新兴产业。积极发展新能源、新材料、生物发酵和装备制造等战略性新兴产业。继续推进华能、大唐、国华、国网、龙源等风电项目和东方电气风力发电机组组装、鸿洋药业生物制药基地等项目开工建设；确保华通海拉尔4000台高低压配电柜5000千米电线电缆、天地公司年产1000台高低压配电柜1万吨空铝绞线项目落地。加快锦化机6000吨压力容器、牙克石北方药业系列抗生素原药和阜丰扎兰屯氨基酸生产基地等续建项目建设进度。三要继续强化工业园区建设。进一步完善园区总体规划，推进园区整合，明确各园区功能定位和产业布局。继续加大园区基础设施建设力度，提升园区承载能力。进一步加大工业园区核心产业和项目的引进培育力度，以重大项目带动产业集聚和产业延伸，带动中小企业配套加工，形成大中小项目协调推进的发展格局。四要全面抓好节能减排工作。单位GDP能耗控制在自治区下达的指标任务以内，加大主要污染物减排力度，开展重点污染源治理，积极淘汰落后产能。

（二）以农牧林业丰收和农畜林产品深加工为重点，繁荣农牧林区经济。高度重视“三农三牧三林”工作。第一产业增加值增长10%，粮食总产量稳定在100亿斤以上，牧业年度牲畜头数稳定在1600万头只以上。一要加强农田水利基础设施建设。认真落实中央1号文件精神，大力加强节水灌溉、小型农田水利重点县和病险水库除险加固等农田水利工程建设。开工建设杨旗山水库和尼尔基下游灌区项目，争取扎敦水利枢纽项目立项。全力推进晓奇水利枢纽、毕拉河水利枢纽、诺敏灌区和晓奇灌区前期工作进程。完成年度中小河流治理任务和安全饮水建设任务。抓好防汛和抗旱工作。实施好百亿斤粮食水利项目和国家坡耕地水土流失综合治理试点工程，全年新增节水灌溉面积35万亩，新增牧区饲草料地节水灌溉面积3.2万亩。二要大力推进农牧业产业化。把提高农畜产品加工转化增值作为推进农牧业产业化的重点，大力扶持具有区域带动作用的大型龙头企业发展，将一产的初级原料尽快向二产转化，延长产业链条，实现加工增值。乳品以调整产品结构、提升产品档次为重点，实现由初级加工向精深加工转变。肉类以牛羊肉、猪肉加工为重点，大力提高肉类精深加工水平，积极发展以冷鲜肉为主的系列产品，加大对畜禽内脏及毛、骨、血等附加产品的综合开发，发展生化制药，达到资源的充分利用。粮油以玉米、大豆等加工为重点，加大加工转化力度，实现转化增值，提高粮油加工产品的市场竞争力。马铃薯要大力发展各种不同工业用途的变性淀粉及衍生物产品，提高加工转化率。三要扎实推进新农牧林区建设。围绕优化农牧林业结构和提升产业水平，全力扩展新农牧林区建设试点样板村(嘎查)，培育主导产业。加大农牧林区基础设施和公共公益设施建设投入，进一步完善农牧林区综合信息服务体系。深入开展“村容整洁美化”工程，改善居住环境。四要多渠道提高农牧民收入。全面落实国家各项惠农惠牧政策，实现政策增收；鼓励农牧民引进新品种和新技术，发展高回报的设施农牧业，实现科技增收；加强农牧民职业技能和创收能力培训，鼓励农民转移就业、返乡创业，实现就业创业增收。

（三）以旅游业为核心，促进服务业大发展。一要推进旅游产业升级。全年旅游接待人数达到1157万人次，增长18%；旅游业总收入完成172亿元，增长20%。继续抓好呼伦贝尔民族文化园、蒙古之源•蒙兀室韦民族文化园、拓跋鲜卑民族文化园规划建设，确保白音哈达草原景区投入运营。全力做好敖鲁古雅使鹿部落景区、达斡尔民族园、东北抗联英雄纪念园等景区的续建工程，积极推进鄂温克风情园、扎兰屯昂勒滑雪小镇等新项目建设进程，着力提升莫尔道嘎、红花尔基、柴河景区等级。大力发展休闲农业旅游，创建全国休闲农业与乡村旅游示范县和全国休闲农业示范点。全面提高接待能力和水平，努力改善旅游交通条件。加强旅游行业管理，规范整顿旅游市场，提高旅游服务质量。以冬季旅游市场开发为重点，借助媒体平台和利用我市特色节庆活动，重点针对俄蒙、东北、京津冀、“长三角”和“珠三角”等客源市场，开展旅游宣传促销和旅游协作。二要加快物流业发展。重点发展口岸国际物流带、滨州干线物流带和岭东、岭西物流区，全力抓好扎兰屯大乾粮食物流中心、海拉尔国安物流等项目建设，积极推进牙克石综合物流产业园、阿荣旗岭东农畜产品交易中心等项目的规划和报批工作，争取早日开工建设。三要加快商业及市场体系建设。加快中心城市商业圈的培育建设，强化服务功能；进一步推进“万村千乡”市场工程和“新网工程”建设，积极争取国家扶持农副产品批发市场和城镇农贸市场建设有关政策和资金，推进全市各类市场升级改造。四要推动金融业发展。加快招商银行、盛京银行等域外金融机构入驻工作，推动内蒙古银行和包商银行向旗市延伸服务网络，大力发展村镇银行等新型农村金融机构。优化金融环境，鼓励金融机构拓宽信贷领域、增加信贷投放。强化信用担保体系、诚信体系和中小企业信用体系建设，构建多层次融资平台。

（四）以提高可持续发展能力为目标，抓好生态和基础设施建设。基础设施建设是推动经济社会持续发展、保障和改善民生的基础，必须要加大投入、全力推进。生态建设要组织实施好天保二期、退耕还林还草、重点公益林保护等工程建设。今年完成100万亩沙区综合治理任务，并对上两年建设成果进行巩固。认真落实《大小兴安岭林区生态保护和经济转型规划》，森林采伐量控制在130万立方米左右。继续加大国家森林城市创建力度，全面加强城市绿化。落实好国家草原生态补助奖励政策，切实做好草原生态保护。编制好林地保护利用规划，统筹协调林地的保护与利用。公路争取完成投资70亿元，力争阿荣旗―博克图―牙克石高速公路、海拉尔―阿木古郎―杜拉尔一级公路一幅建成通车，争取新林北―扎兰屯高速公路、根河―拉布大林、金界壕―大兴、满洲里―阿拉坦额莫勒―阿木古郎一级公路开工建设，加快推进海拉尔—拉布大林、那吉屯—尼尔基、阿里河—加格达奇一级公路、黑山头—室韦边防口岸公路及其他县乡路网项目前期工作，全面实施通村油路工程。铁路重点加快推进阿荣旗—扎兰屯地方铁路建设，全面完成海拉尔火车站改造工程，加快海拉尔—黑山头、阿荣旗—讷河等铁路前期工作。继续加大扎兰屯、根河等五个民航支线和通勤机场前期工作力度，完成呼伦贝尔海拉尔机场停机坪扩建工程。

（五）以做大做强中心城区为突破口，加快城镇化进程。一要抓好城市规划。全面抓好《呼伦贝尔市城市总体规划》修编和中心城详细规划的编制及设市城市、城关镇的控制性详细规划的编制工作，实现城乡规划全覆盖。二要做大做强中心城市。加快推进呼伦贝尔中心城区建设步伐，按照承载60万人口的规模，统筹抓好海拉尔区、鄂温克旗巴彦托海镇和陈巴尔虎旗宝日希勒镇发展。进一步完善中心城新区管理体制，加大企业区域总部和生活基地建设。全面启动中心城新区河东部分建设，高标准建设好2.2平方公里中心文化区。继续推动满洲里、牙克石、扎兰屯等区域性中心城市和旗市所在地城关镇建设。三要加大城市公共基础设施建设力度。加快城市给排水、供热供气、城镇道路、污水处理和垃圾无害化处理等基础设施建设。四要着力推进移民扩镇工程。按自愿、依法、有序的原则，稳妥地推进农牧民和林区群众向城镇转移，有计划、有步骤地解决农牧民工和林区群众在城镇的就业和住房等问题。五要继续实施保障性安居工程。建设保障性住房7万套370万平方米，其中新建廉租房6364套30万平方米、公租房2200套13.2万平方米、棚户区改造5.5万套290万平方米。

（六）以增强经济社会发展活力为方向，深化改革扩大开放。一要扎实推进各项改革。积极争取将我市整体列入国家《资源型城市可持续发展规划》范围。进一步完善土地草牧场承包经营权流转。深化集体林权制度改革，确保年底前完成主体改革任务。大力推进文化领域资源整合，促进文化与旅游产业相结合。加快推行事业单位分类改革和绩效工资制度改革。继续支持森工、农垦等国有大企业推进改革。二要全面扩大开放。加强与俄蒙的经贸往来和教育、文化、卫生等方面的人文交流，支持满洲里国家重点开发开放试验区建设，带动全市口岸经济全面发展。积极争取将呼伦贝尔整体纳入国家开发开放试验区，并将绥满开放经济带上升为国家发展战略。推进《中俄合作纲要》的实施，加强与俄蒙在矿产资源开发、木材加工、农业生产等领域的经济技术合作，积极推进俄罗斯诺永达拉果铅锌矿、别列佐夫铁矿和蒙古国乌兰铅锌矿等境外资源开发项目建设，扩大进口资源落地加工规模。全年对外贸易进出口总额完成28亿美元，增长20%；口岸过货量达到3000万吨，增长7%。三要加强区域合作。加强与东北三省联合与协作，健全地市、旗县和部门、行业间的协调协作机制，推进吉林天富城市燃气、辽宁沈煤大型煤电基地、与黑龙江省联动发展煤化工等项目进程。加大招商引资工作力度，主动承接东北、京津冀、长三角、珠三角等发达地区产业转移，尽快构筑大范围配置资源的开放格局。全年引进市外（国内）资金558亿元。四要大力扶持非公经济发展。进一步研究制定扶持非公经济发展的政策措施，鼓励引导非公经济围绕优势特色产业搞延伸，围绕重点项目搞协作，围绕大型基地搞配套。把培育壮大本土企业与引进外阜企业同谋划、同部署、同扶持，提升本土企业发展竞争力。积极做好本土企业上市的前期准备工作。

（七）以保障和改善民生为落脚点，发展各项社会事业。各级政府要切实承担起保障和改善民生的责任，努力推动幸福呼伦贝尔建设。切实抓好高校毕业生、农民工就业和困难弱势群体的就业援助工作，城镇新增就业达到3.15万人，农村牧区劳动力转移就业达到7.5万人。认真抓好国家和自治区兴边富民重点县各项工作，积极配合自治区有关厅局，做好对鄂伦春旗和莫旗的帮扶工作。精心组织实施整村推进和产业化扶贫等工程，解决11万贫困人口温饱问题。督促企业构建合理公正的分配制度，落实最低工资标准，逐步提高劳动者工资水平。扩大新型农村牧区社会养老保险试点范围，参保人数达到25万人。提高城乡低保、“五保”供养、城镇“三无”人员、孤儿、养老等困难群体补助标准，同步提高各类弱势群体生活水平。加强物价监管，维护市场秩序，防止通货膨胀。全面实施素质教育，更加注重民族教育和校外教育，促进各级各类教育协调发展，加快推进中小学校舍安全工程建设；全部免除用蒙语授课的高中学生、中等职业教育学生、普通高中家庭经济困难学生的学费和教科书费，三年内全部实现高中免费教育。深入推进医药卫生体制改革，巩固和扩大新型农村牧区合作医疗覆盖面，参合率稳定在90%以上。在公办基层医疗卫生机构全面实施国家基本药物制度，积极探索公立医院改革，进一步完善基层医疗卫生服务体系，促进基本公共卫生服务逐步均等化。扶持蒙中医药事业健康发展。强化地方政府的监管责任，加强疾病预防控制和食品药品安全监管。建立健全技术创新体系和科技服务体系，继续推进科技特派员创业工程，加强科普宣传和科技成果转化，深入推进知识产权战略实施，构建优势产业技术创新战略联盟。加快发展文化事业，实施文化信息资源共享、苏木乡镇综合文化站等五大公共文化服务工程，加强文物和非物质文化遗产保护；继续打造呼伦贝尔五张文化名片，积极推进呼伦贝尔艺术馆和图书馆等工程建设；进一步加快文化产业发展，成立市民族演艺集团。加强体育基础设施建设，广泛开展全民健身运动。强化人才开发利用，统筹推进人才队伍建设，落实好“三支一扶”等选拔高校毕业生到基层工作计划，做好创新型高层次人才和农牧林区人才资源开发等工作，积极推进人力资源市场建设。全面抓好广播影视工作，启动20户以下自然村“村村通”工程，完成广播电视无线覆盖工程任务。切实加强人口和计划生育工作，稳定适度低生育水平，提高出生人口素质。进一步加强乡镇基础设施建设，改善基层干部工作生活条件。重视发展公益慈善事业，全面做好残疾人保障和服务体系建设。加强社区建设与管理，高质量按期完成社区办公场所建设。积极做好老龄工作。落实民族宗教政策，认真做好民族宗教工作。统筹抓好统计、审计、国土、气象、地震、质监、档案史志和外事侨务等工作。

今年继续为群众办10件实事：一是建设1024户老年公寓，逐步探索解决老年人社会化养老问题；二是关爱老年人，对全市65岁以上老年人免费提供健康教育与体检，惠及20.2万人；三是实施草原畜牧业重大自然灾害防控体系建设项目，集中进行牧业四旗饲草料储备和破雪开路机械购置，增强草原畜牧业防灾抗灾能力；四是增加民族教育发展投入，安排民族教育发展专项基金300万元，改善民族教育办学条件，提升办学水平；五是实施市本级广播林区覆盖工程，使市广播电台汉语广播节目覆盖到所有林业旗市，惠及40万人；六是实施农村牧区安全饮水工程，投资1.14亿元，解决10万人安全饮水问题；七是启动呼伦贝尔平安城市及智能交通管理系统建设，为进一步打造平安和谐的社会环境提供有力保障；八是实施农村牧区危房改造项目，改善3000户农牧民居住条件，惠及近万人；九是实施农业生产保险补贴项目，促进粮食生产安全和农民稳定增收；十是加大对呼伦贝尔学院投入，改善办学条件，提高教育质量，顺利实现本科验收。继续实施好市精神卫生中心、海拉尔火车站改造及站前环境整治、艺术馆、科技馆、城市规划展览馆等23个跨年度公共公益工程，力争尽快投入使用。

（八）以推动社会全面进步为着力点，加强精神文明、民主法制、社会管理与创新和政府自身建设。全面加强精神文明建设。进一步加强和完善思想道德建设，持之以恒地加强社会主义精神文明建设和社会主义核心价值体系建设，坚持正确导向，唱响主旋律，继续提高舆论引导能力。广泛开展群众性精神文明创建活动，全力办好首届全市精神文明建设经验交流会。进一步提升公民文明素质和社会文明程度，增强社会诚信。继续抓好双拥模范城创建工作，着力提升全市精神文明建设水平。

全面加强民主法制建设。认真执行人大及其常委会的各项决议、决定，自觉接受监督。积极支持政协履行政治协商、民主监督、参政议政的职能。认真办理人大代表议案、建议和政协委员提案，主动听取各民主党派、工商联、无党派人士及各人民团体的意见和建议。大力支持工青妇等群团组织开展工作。深入开展普法教育，做好法律援助工作。

全面加强社会管理与创新。最大限度地激发社会活力，最大限度地维护社会和谐，着力提高社会管理科学化水平，促进社会公平正义。建立健全应急管理体制机制，提高预防和处置各种自然灾害及城镇、森林草原火灾等突发公共事件能力。深入开展平安创建活动，严格落实维稳工作责任制，全面加强社会治安综合治理，严厉打击违法犯罪活动。高度重视信访工作，认真解决信访突出问题，积极做好群体性事件预防处置，确保社会稳定。

全面加强政府自身建设。政府的权力是人民赋予的，必须对人民负责，为人民服务，接受人民监督，努力建设人民满意政府。一要努力建设高效政府，进一步深化政府机构改革，加快形成权责一致、分工合理、决策科学、执行顺畅、监督有力的行政管理体制。大力简化办事程序，精简和规范审批事项，高度重视和加强行政审批中心建设，进一步完善功能，逐步推行网上审批、全程办事代理和行政效能电子监察，切实提高政府工作效能。二要努力建设阳光政府，大力推进政务公开，重点推进财政预算、公共资源配置、重大项目建设、社会公益事业建设等领域的信息公开。加快完善电子政务系统，畅通政府与群众双向沟通渠道，确保广大人民群众的知情权和参与权，使政府工作置于人民群众的监督之下，让权力在阳光下运行。三要努力建设法制政府，大力推进依法行政，始终把依法行政贯穿于政府决策、执行、监督的全过程，严格依照法定权限、程序和职责行使权力、履行职责、规范行为。从严落实行政执法责任制和执法过错追究制，切实解决不作为和乱作为的问题，促进公正执法。四要努力建设廉洁政府，全面加强廉政建设，严格执行党风廉政建设责任制和领导干部廉洁从政准则，强化行政监察和审计监督，严格责任追究和行政问责，切实加大反腐倡廉力度。各级干部尤其是领导干部要牢记使命，廉洁从政，以身作则，带动政风行风进一步好转。

各位代表，回首过去，我们创造了不平凡的业绩；展望未来，前景美好，责任重大。让我们在自治区党委、政府和市委的坚强领导下，团结带领全市各族人民，解放思想，扎实工作，为实现呼伦贝尔“美丽发展、科学崛起、共享繁荣”的宏伟目标而努力奋斗！
